# Annotations

**Published:** 1901-09-28

**Authors:** 


### ANNOTATIONS.

### "Substitution" in Dispensing.

Whether or not we in England are living in a fool's paradise in regard to the purity and strength of the medicines which we trustingly prescribe for our patients and which our patients even more trustingly obtain from their chemists we do not know. We have faith; we place our trust in our friends the chemists: the whole affair is a matter of confidence, and as a rule we do not think that our confidence is misplaced. Every now and again, however, even in this good and respectable England of ours, things happen which raise uncomfortable doubts; and this at least we may say, that the frequency with which patients complain that prescriptions come out differently at the hands of different dispensers, forces us to suspect that all chemists do not stick so closely as they should do to the Pharmacopœia. In America, "substitution" appears to be almost a trade, and it seems to us that if one-half of what we hear about it is true, the medical profession will soon be driven to return to the old custom of dispensing its own medicines. When we are told in reputable American papers that there are in that country large manufacturing concerns which put up nothing but imitation medicines and drugs; that non-officinal leaves are ground up with those which meet the requirements of the Pharmacopœia; that tinctures are made from fluid extracts instead of from fresh drugs; and that old, weather-beaten drugs are sold in the open market for use in the preparation of potent remedies, or rather of remedies which are imagined to be potent, we cannot but wonder whether anything of the same kind is taking place here, and whether the calm confidence with which we entrust our patients' lives to the more or less accidental circumstances which determine the choice of a chemist is quite justifiable. We often find medical men protesting against the increasing practice of prescribing ready-made medicines and other proprietary articles which are sold in "original packages" protected by trade marks, and under names which are registered to prevent substitution. Those who limit their prescriptions to such preparations lose the advantage of being able to make slight alterations of dose to fit individual cases, and we may be sure that this growing system of machine-made medication would not have obtained the position it now occupies had there not been some very solid reason for it. The reason is the fear of substitution, and the confidence given by the "original package" that, whether it be bought at the Land's End or at John o' Groats, the contents will be identical.

### Self-Sacrifice in the Cause of Science.

In view of the fact that from both sides of the Atlantic there have come offers to submit to experiment with the object of testing the question of the infectivity of bovine tuberculosis to man, it may be well to say that the question is not one which is likely to be answered off-hand in the manner suggested. The men who have made these offers are the heroes of the hour, and we quite agree with Dr. Garnault that "on the battlefield of social existence a man, fully conscious of his acts, can well do as much as so many do on real battlefields." Courageous self-sacrifice for the good of others is fully deserving of all honour. At the same time it is as well to bear in mind that the problem is not thus to be solved. If the result of the experiments were negative, of course nothing would be proved one way or another, for we know perfectly well, even in regard to diseases as to the infectivity of which there is no possible doubt, how often infection may fail to "take;" while if the result were positive an isolated instance would be as nothing in the decision of so great a question. In experimenting with a disease the germs of which are so widely spread as are those of tuberculosis the chances of error are too great. Clearly the first thing to do is to find out by experiment on the large scale whether human tuberculosis is so different a thing from bovine as to be incapable of producing the latter disease in cattle. If that cannot be proved the whole thing falls to the ground and we are as we were again. If, however, it should be shown that human tuberculosis really is so distinct from bovine that it is incapable of producing the latter disease in cattle we should then come to the question whether, even so, the infection can be transferred the other way, namely, from cattle to man, and that is a problem which, as we have said, cannot be decided by a few isolated experiments on human beings who are all the time exposed to infection from other sources. We must not use up good heroes in performing inconclusive experiments.

### The Examination of Recruits.

The frequent complaints from South Africa in regard, to the quality of the reinforcements despatched from England, and the fact that the blame for "passing" these inefficient soldiers as fit for active service is at once thrown upon the doctors, remind one of the old Biblical adage about the labourer being worthy of his hire, and lead one to question whether there may not be truth in the natural corollary that the value of the work done has some relation to the recompense which the labourer receives While, therefore, we would not pretend to excuse carelessness or bad work, we would insist that the Government department by which the fees paid for the medical examination of recruits are fixed is quite as worthy of blame as the practitioners who in return for a most inadequate fee have given an inadequate examination. The generally accepted fee for an examination for life assurance is one guinea, and although certain offices choose only to pay half a guinea when the sum assured is of small amount, it is pretty well recognised that they risk their stability by so doing. Life assurance, however, is a mere matter of business in which risks have to be balanced one against the other. But the risks of war are not mere matters of business. The first object of the war is to get it over as soon as possible, and every breakdown from sickness or inefficiency not only involves the loss of all that it has cost to train the soldier and carry him to the seat of operations, and all that it costs in money and in men to cure him or return him home again, but in many cases imperils the lives of his companions and upsets the whole plan of a campaign, the mere money cost of which runs into hundreds of millions of pounds. The proper physical examination of recruits for which the doctor obtains the magnificent remuneration of half-a-crown is then infinitely more important even from a mere money point of view than is the examination for life assurance, for the proper performance of which the best and most carefully-managed offices in the country are glad to give a guinea, and in some cases give more. The examination for the militia is, we believe, often done at a still cheaper rate, and although we would not wish to excuse bad work, those who fix these rates should certainly share the blame if the labourer can be shown by his bad work to have been only worthy of the small hire offered to him.

